# Divergent soil filtering of leaf functional traits of *Pinus yunnanensis* in non-karst and karst habitats: from pH screening to stoichiometric control

**DOI:** 10.3389/fpls.2026.1833651

**Published:** 2026-05-19

**Authors:** Bin He, Wangjun Li, Xiaolong Bai, Tu Feng, Shun Zou, Qing Li

**Affiliations:** College of Ecological Engineering, Guizhou University of Engineering Science, Bijie, Guizhou, China

**Keywords:** environmental filtering, karst habitat, leaf functional traits, phenotypic integration, *Pinus yunnanensis*

## Abstract

**Introduction:**

Karst ecosystems impose severe edaphic stresses, particularly phosphorus limitation, yet how these filters reshape plant phenotypes through altering trait coordination remains unclear. This study aimed to elucidate how karst habitats reshape the functional phenotype of *Pinus yunnanensis* by examining leaf trait shifts and coordination patterns.

**Methods:**

We compared key leaf functional traits between paired karst and non-karst sites. An integrated analytical framework was applied, including correlation analysis, network analysis, redundancy analysis (RDA), and partial least squares structural equation modeling (PLS-SEM) to identify trait variation patterns and their environmental drivers.

**Results:**

*P. yunnanensis* in karst habitats exhibited a resource-conservative strategy, with significantly lower specific leaf area and leaf phosphorus concentration, but higher leaf thickness, leaf dry matter content, and leaf nitrogen to phosphorus ratio. Network analysis revealed a fundamental reorganization of trait relationships: karst habitats formed a tightly connected network centered on stoichiometric traits, whereas non-karst habitats displayed an SLA-centered architecture. RDA identified soil phosphorus availability and stoichiometric ratios as primary drivers in karst habitats, while soil pH and nitrogen availability predominated in non-karst habitats. PLS-SEM indicated that karst environments attenuated the trade-off between leaf morphological and chemical traits.

**Conclusion:**

Karst environments function as potent biogeochemical filters that not only select for conservative trait values but also fundamentally reconfigure phenotypic integration. The architectural shift from a resource-acquisitive to a persistence-oriented phenotype demonstrates how extreme environments reshape plant adaptation through altered trait coordination networks.

## 1 Introduction

Karst regions represent a critical and ecologically vulnerable component of the Earth’s Critical Zone, characterized by a binary hydrogeological structure arising from the dissolution of carbonate rocks (Wang et al., 2017). This structure results in scarce surface water and deeply embedded groundwater (Xiong et al, 2012), imposing persistent and intense drought stress on local vegetation (Liu et al., 2011). Globally, karst landscapes cover approximately 15% of the continental area and support nearly 25% of the world’s population (Hartmann et al., 2014). Among these, the karst region in southwestern China is the world’s largest contiguous karst area (Wang et al., 2003). Under increasing human activities and climate change, these regions face severe soil erosion, water loss, and rocky desertification (Xu et al., 2013), threatening ecosystem stability (Liu et al., 2005). Consequently, ecological restoration in these areas is a strategic priority for China’s ecological security and sustainable development.

Vegetation restoration, particularly afforestation, is a fundamental strategy to control rocky desertification. A critical prerequisite is identifying tree species adapted to the unique karst habitat. However, a major shortcoming of current research is the limited understanding of the specific adaptation strategies and underlying mechanisms of karst-adapted plants (Liu et al., 2025). In particular, it remains unclear which leaf functional traits are most responsive to karst stress and how they mediate plant adaptation.

Plant functional traits reflect strategic trade-offs inherent in resource acquisition and allocation across different organs, providing critical insights into ecosystem structure and function (Díaz and Cabido, 2001; McGill et al., 2006). Among plant organs, leaves are especially important as the primary sites of photosynthesis and energy exchange (Ackerly et al., 2002). Specifically, leaf morphological traits (e.g., leaf area, specific leaf area, leaf dry matter content) and elemental composition (e.g., carbon, nitrogen, phosphorus) jointly determine photosynthetic efficiency and resource-use costs (Wright et al., 2004). Consequently, these traits serve as reliable proxies for characterizing plant ecological strategies and resource-use patterns (Kleyer et al., 2019; Zhang et al., 2012). The correlations among these traits, largely governed by evolutionary constraints, constitute the theoretical framework of the leaf economics spectrum (Wright et al., 2004; Firn et al., 2019). While recent studies emphasize the necessity of integrating multiple trait dimensions to decipher plant responses to compounded stresses—such as drought and nutrient limitation (Yi et al., 2022; Li et al., 2024)—further inquiry is essential to unravel the complex mechanisms linking leaf functional traits to environmental drivers across contrasting habitats.

Comparative analysis of leaf functional traits between dominant species from contrasting habitats offers valuable insights into adaptive strategies (Geekiyanage et al., 2019). Karst and non-karst habitats differ markedly in soil properties — such as pH, water availability, and calcium concentration — which profoundly influence plant growth (Hao et al., 2015; Kinzel, 1983). Previous studies indicate that karst tree species generally exhibit higher leaf dry matter content and elevated concentrations of leaf nitrogen, potassium, calcium, and magnesium, along with reduced leaf area and specific leaf area compared to non-karst species (Tang et al., 2021; Yang et al., 2020), suggesting a conservative resource-use strategy. Nevertheless, a centrally unresolved question is whether intrinsic plant characteristics or external environmental factors play a more dominant role in shaping leaf functional traits under such heterogeneous conditions. Moreover, it is unclear whether the drivers of trait variation differ substantially between karst and non-karst environments. Addressing these knowledge gaps is crucial for guiding species selection in restoration projects aimed at mitigating rocky desertification.

In southwestern China, the coexistence of karst and non-karst landscapes under comparable climatic conditions provides a unique natural laboratory to examine how lithology shapes plant adaptation. This study focuses on *Pinus yunnanensis*, a species distributed across both habitat types in northwestern Guizhou Province. We analyzed leaf morphological and elemental traits to elucidate the mechanisms underlying plant adaptation to contrasting habitats. Specifically, this study addresses the following questions: (1) How do leaf functional traits differ between karst and non-karst populations of *P. yunnanensis*? We hypothesize that greater abiotic stress in karst habitats selects for higher leaf nutrient concentrations, smaller leaf area, and lower specific leaf area. (2) How do trait relationships differ between karst and non-karst habitats? We anticipate stronger correlations between leaf nutrients and morphological traits in karst environments, reflecting more integrated resource-use strategies. (3) What are the primary environmental drivers governing trait variation in each habitat? We hypothesize that distinct soil properties (e.g., calcium availability and phosphorus limitation) lead to different key factors influencing trait expression between the two habitats.

## 2 Materials and methods

### 2.1 Study sites

**Figure 1:**
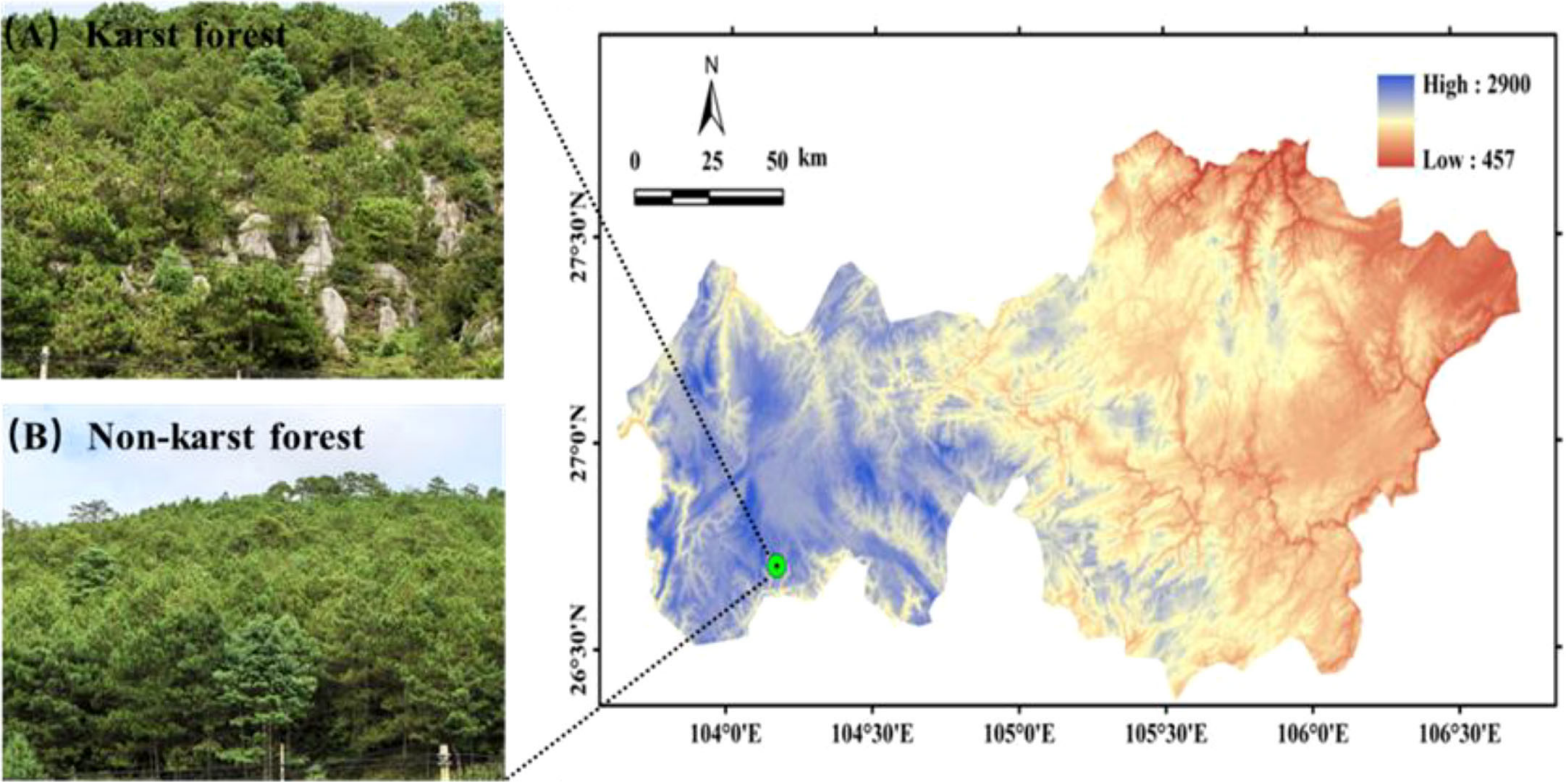
Geographical location of the study area in Bijie City, Guizhou province, Southwest China.

This study was conducted in Weining County (103°36′-104°45′ E, 26°36′-27°26′ N) (**Figure 1**), located in the northwestern part of Guizhou Province, China. The area is characterized by a typical karst plateau landscape. The climate is classified as a subtropical plateau humid monsoon type, featuring modest annual temperature fluctuations but pronounced diurnal temperature variations, distinct dry and wet seasons, and synchronous patterns of rainfall and heat. The mean annual temperature is 10.5 °C, with recorded extremes ranging from -15.3 °C to 31 °C. Annual precipitation averages 960–1100 mm, coupled with 1700–1945 hours of sunshine and a frost-free period of approximately 195 days (Wang et al., 2024). The region exhibits contrasting lithologies that give rise to two distinct soil types. In the karst habitat, soils are classified as rendzic leptosols (or calcareous lithosols according to the Chinese Soil Taxonomy), which are shallow, calcium−rich soils directly overlying limestone bedrock. In the non−karst habitat, soils are haplic alisols (equivalent to yellow−brown earths in the Chinese classification), derived from sandstone and basalt parent materials. Native vegetation is composed of plateau mountain evergreen broad-leaved forests, coniferous forests, shrubs, and meadows. The coniferous forests are predominantly occupied by *Pinus yunnanensis*, *Pinus armandii*, and *Cunninghamia lanceolata*. Historical intensive human activities, particularly excessive land reclamation, have led to significant vegetation degradation and increased the fragility of the local ecosystem. In response, large-scale ecological restoration projects, such as the National Rocky Desertification Control and Grain for Green Program, have been implemented in recent decades. These initiatives involve practices like afforestation, grassland restoration, and mountain closure, aiming to facilitate the recovery of the ecological environment.

### 2.2 Experimental design

To assess the influence of habitat type on forest ecosystems, a habitat-comparison design was employed within the core distribution zone of *P. yunnanensis*. The study targeted paired karst and non-karst habitats. In each habitat type, five replicate plots (20 m×20 m) were systematically established (n = 5 per habitat, total N = 10). Critically, plot locations were meticulously selected to ensure precise pairwise matching of key topographic variables-slope gradient and slope position-between the two habitat types. This design explicitly controls for the confounding influence of topography, thereby allowing a more direct attribution of observed differences to the underlying habitat (karst vs. non-karst). A comprehensive summary of the abiotic and biotic characteristics for all plots is presented in **Table 1**.

**Table 1:** General characteristics of the sample plots across karst and non-karst forest habitats.

Habitats	Plantation	Forest age	Altitude /m	Slope/(°)	Slope aspect	Average tree height/m	Average diameter at breast eight/cm
Karst	*Pinus yunnanensis*	21	2207	12	Northeast	12	13.2
Non-karst	*Pinus yunnanensis*	21	2205	9	Northeast	15	14.5

### 2.3 Sample collection and processing

Plant and soil sampling was conducted from July to August 2024. Leaf sampling followed standard protocols for the assessment of leaf functional traits (Pérez-Harguindeguy et al., 2013). In each plot, three healthy *P. yunnanensis* trees with similar diameter at breast height and without visible canopy damage were randomly selected. From each tree, current-year branches were collected from the mid-outer canopy layer in all four cardinal directions using a pole pruner. Thirty bundles of healthy, undamaged current-year needles were selected from each branch. The needles were immediately wrapped in moist filter paper, placed in cooled containers, and stored at 4 °C for subsequent morphological measurements. Additionally, a separate set of needle samples was randomly collected, placed in paper bags for the determination of carbon (C), nitrogen (N), phosphorus (P) and calcium (Ca) concentrations. Samples from each individual tree represented one biological replicate (n = 3 per plot).

Soil samples were collected concurrently with leaf sampling using a five-point sampling method (four vertices and center of each plot). After removing the surface litter layer, soil cores (3.5 cm diameter) were taken from 0–20 cm depth at each sampling point. Cores from the same plot were combined to form a composite sample, sieved through a 2-mm mesh to remove roots and debris, and stored in sealed plastic bags at 4 °C for subsequent analysis of soil chemical properties. Three independent composite samples were collected and analyzed per plot.

### 2.4 Measurement of plant functional traits

The twelve functional traits evaluated in this study are listed in **Table 2**. Needle samples were equilibrated at 5 °C for 12 h in darkness prior to analysis. After surface-drying, leaf fresh weight (LFW) was measured on an analytical balance (± 0.001 g). Leaf area (LA) was determined from digital images acquired with a flatbed scanner and processed using ImageJ software (National Institutes of Health, USA). Leaf thickness (LT) was recorded as the mean of three measurements taken at the base, middle, and apex of each needle with a digital vernier caliper (± 0.02 mm). For dry weight determination, samples were first heated at 105 °C for 30 min to deactivate enzymes and then dried at 80 °C until a constant weight to obtain leaf dry weight (LDW). The specific leaf area (SLA) and leaf dry matter content (LDMC) were calculated as follows:

**Table 2:** Leaf functional traits measured in this study, including their names, abbreviations, and units.

Trait category	Trait name	Abbreviation	Unit
Leaf Morphology	Leaf Length	LL	cm
	Leaf Thickness	LT	mm
	Leaf Area	LA	cm^2^
Leaf Economics	Specific Leaf Area	SLA	cm²·g^-^¹
	Leaf Dry Matter Content	LDMC	mg·g^-^¹
Leaf Elemental Composition	Leaf Carbon Content	LCC	g·kg^-^¹
	Leaf Nitrogen Content	LNC	g·kg^-^¹
	Leaf Phosphorus Content	LPC	g·kg^-^¹
	Leaf Calcium Content	LCaC	g·kg^-^¹
Stoichiometric Ratios	Leaf C/N Ratio	L_C:N_	–
	Leaf C/P Ratio	L_C:P_	–
	Leaf N/P Ratio	L_N:P_	–


$$\text{SLA}=\frac{LA}{LDW}$$

$$\text{LDMC}=\frac{LDW}{LFW}$$

Dried leaf material was ground using a ball mill and sieved through a 0.5-mm mesh for elemental analysis. Leaf nitrogen (LNC) and phosphorus (LPC) concentrations were measured colorimetrically with a SEAL AA3 Continuous Flow Analyzer (SEAL Analytical, Germany). Leaf calcium concentration (LCC) was measured by inductively coupled plasma optical emission spectrometry (ICP-OES; iCAP 7400, Thermo Fisher Scientific, USA).

### 2.5 Determination of soil chemical properties

Soil physicochemical properties were determined using standardized methods (Bao, 2000). Soil pH was measured potentiometrically in a 1:2.5 (w/v) soil-to-water suspension. Soil organic carbon (SOC) content was determined by the potassium dichromate oxidation method with external heating. For the determination of total nitrogen (TN), total phosphorus (TP), and total potassium (TK), soil samples were digested with a mixture of sulfuric acid and perchloric acid (H_2_SO_4_-HClO_4_). The TN and TP concentrations in the digestates were then measured using a fully automated chemical analyzer (Smartchem 200, AMS, Italy). The TK concentration was determined by flame atomic absorption spectrometry (FAAS).

Soil available nutrients were extracted and analyzed as follows: available nitrogen (AN) was extracted with 2 mol/L potassium chloride (KCl) and quantified using a continuous flow analyzer; available phosphorus (AP) was extracted with 0.5 mol/L sodium bicarbonate (NaHCO_3_, pH 8.5) and determined by the molybdenum blue colorimetric method; available potassium (AK) was extracted with ammonium acetate (NH_4_OAc) and measured by flame photometry. Exchangeable calcium (Ca^2+^) was extracted using an EDTA-ammonium acetate solution and its concentration was quantified by inductively coupled plasma optical emission spectrometry (ICP-OES; DUO 7400, Thermo Fisher Scientific, USA).

### 2.6 Statistical analyzes

All data were examined for normality and homoscedasticity prior to analysis; non-normal variables were log−transformed. Differences in leaf functional traits between karst and non−karst habitats were assessed using one−way ANOVA with Duncan’s post−hoc test (R package “vegan”). Pairwise trait correlations within each habitat were quantified by Pearson’ correlation coefficients, and relationships between leaf traits and soil properties were explored via redundancy analysis (RDA).

Leaf trait networks (LTNs) were constructed for each habitat using the”igraph”package, with nodes representing traits and edges defined by significant Pearson correlations (|r| > 0, *p< 0.05*). Network topology was characterized by diameter, average path length, and edge density. Bootstrap resampling (1000 iterations) was performed to derive 95% confidence intervals for each metric from the 2.5th and 97.5th percentiles of the bootstrap distributions, thereby strengthening inferential reliability. Differences between habitats were assessed using Tukey’s HSD test (package “multcomp”). Node degree centrality identified hub traits (Kleyer et al., 2019).

Partial least squares structural equation modeling (PLS−SEM) (package “lavaan”) was used to evaluate direct and indirect habitat effects on leaf traits. Traits were grouped into two latent constructs—morphological and chemical—based on their distinct functional roles. *A priori* model was specified with habitat as an exogenous variable directly influencing both latent constructs as well as selected individual traits that exhibited significant inter−habitat differences. The full initial model included all hypothesized direct and indirect pathways among the latent variables. Model simplification was achieved by sequentially removing non−significant paths (*p > 0.05*) to derive a parsimonious final model. Goodness−of−fit was assessed using the following criteria: χ²/df ≤ 2, Comparative Fit Index (CFI)≥0.95, and Root Mean Square Error of Approximation (RMSEA)≤0.05. Statistical significance was set at α = 0.05, and all analyzes were conducted in R version 4.1.2.

## 3 Result

### 3.1 Differences in leaf functional traits of *P. yunnanensis* between karst and non-karst habitats

**Figure 2:**
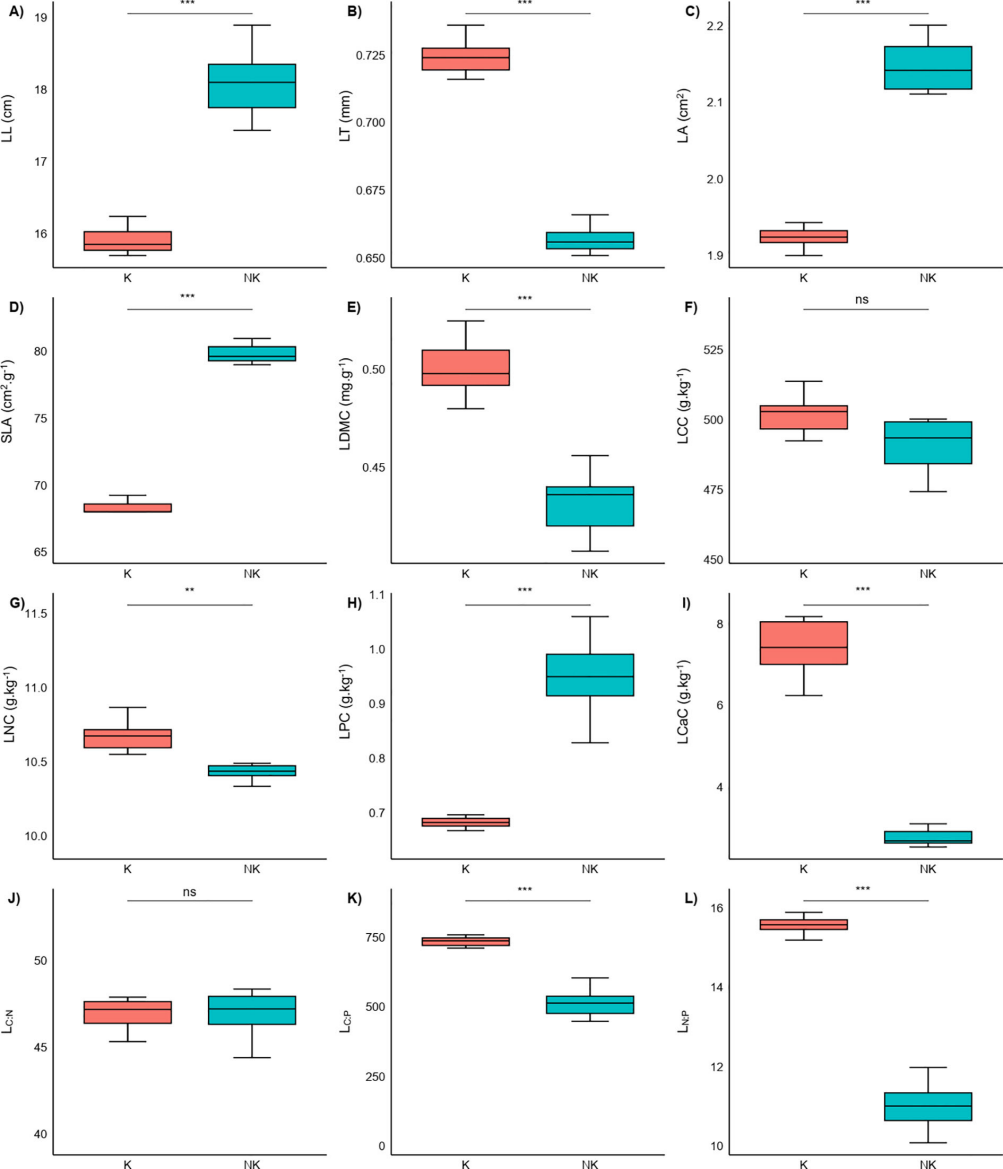
Differences of leaf functional traits between karst (K) and non‑karst (NK) habitats. Boxes in each boxplot **(A–L)** show the first and third quartiles and the median. The upper and lower whiskers indicate the largest and smallest values away from 1.5×IQR (interquartile range) of the third quartiles and first quartiles, respectively. ** p<0.01; *** p<0.001; ns, not significant.

Comparative analysis revealed significant divergence in the leaf functional traits of *P. yunnanensis* across karst and non-karst habitats (**Figure 2**). Foliage from karst habitats exhibited significant reductions (*p<0.05*) in LL, LA, SLA, and LPC. Conversely, these leaves displayed significant increases (*p<0.05*) in LT, LDMC, LNC, LCaC, as well as L_C:P_ and L_N:P_. In contrast, neither leaf carbon content (LCC) nor the leaf carbon to nitrogen ratio (L_C:N_) showed statistically significant differences between the two habitat types (*p>0.05*).

### 3.2 Variation in leaf trait correlations across habitats

**Figure 3:**
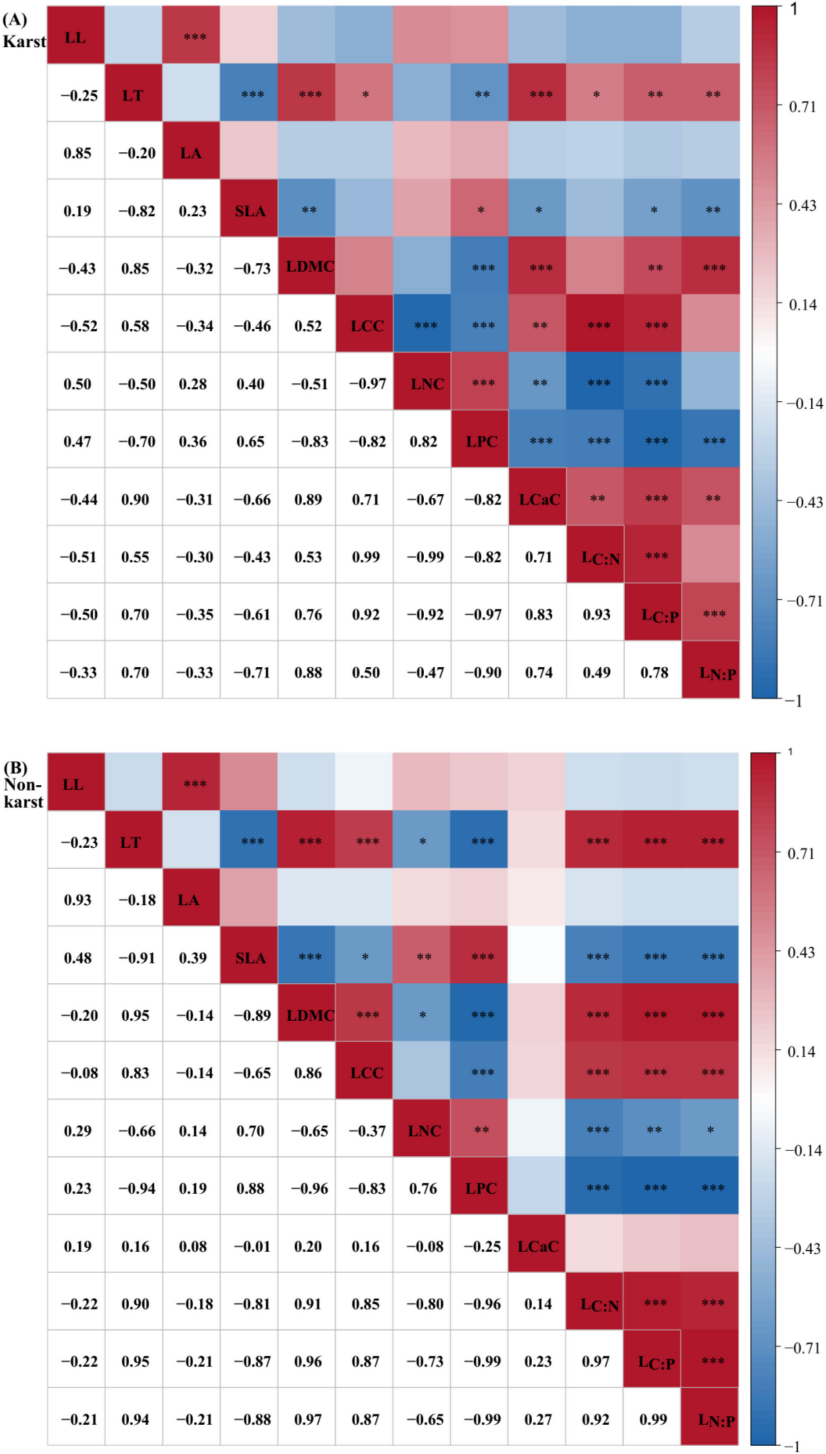
Correlation analysis of leaf functional traits in *P. yunnanensis* across different habitats. The matrix displays Pearson correlation coefficients (indicated by numbers and color gradients), revealing coordinated variation among the traits. Significance levels are denoted by asterisks (*p<0.05; **p<0.01; ***p<0.001).

Distinct correlation patterns of leaf functional traits in *P. yunnanensis* were observed between karst and non-karst habitats (**Figure 3**). In karst habitats, LCaC was positively correlated with LT, LDMC, LCC, L_C:N_, L_C:P_, and L_N:P_ (*p<0.01*), but negatively correlated with SLA, LNC, and LPC (*p<0.05*). Additionally, LCC was negatively correlated with LNC (*p<0.01*), while a positive correlation was detected between L_C:N_ and L_N:P_ (*p<0.05*). In contrast, within non-karst habitats, LCaC was not significantly correlated with any other measured traits. Instead, LCC correlated negatively with SLA and positively with both LDMC and L_N:P_ (*p<0.01*). Similarly, LNC correlated negatively with LT, LDMC, and L_N:P_, and positively with SLA (*p<0.01*). L_C:N_ also showed positive correlations with LDMC and L_N:P_, and a negative correlation with SLA (*p<0.01*).

**Figure 4:**
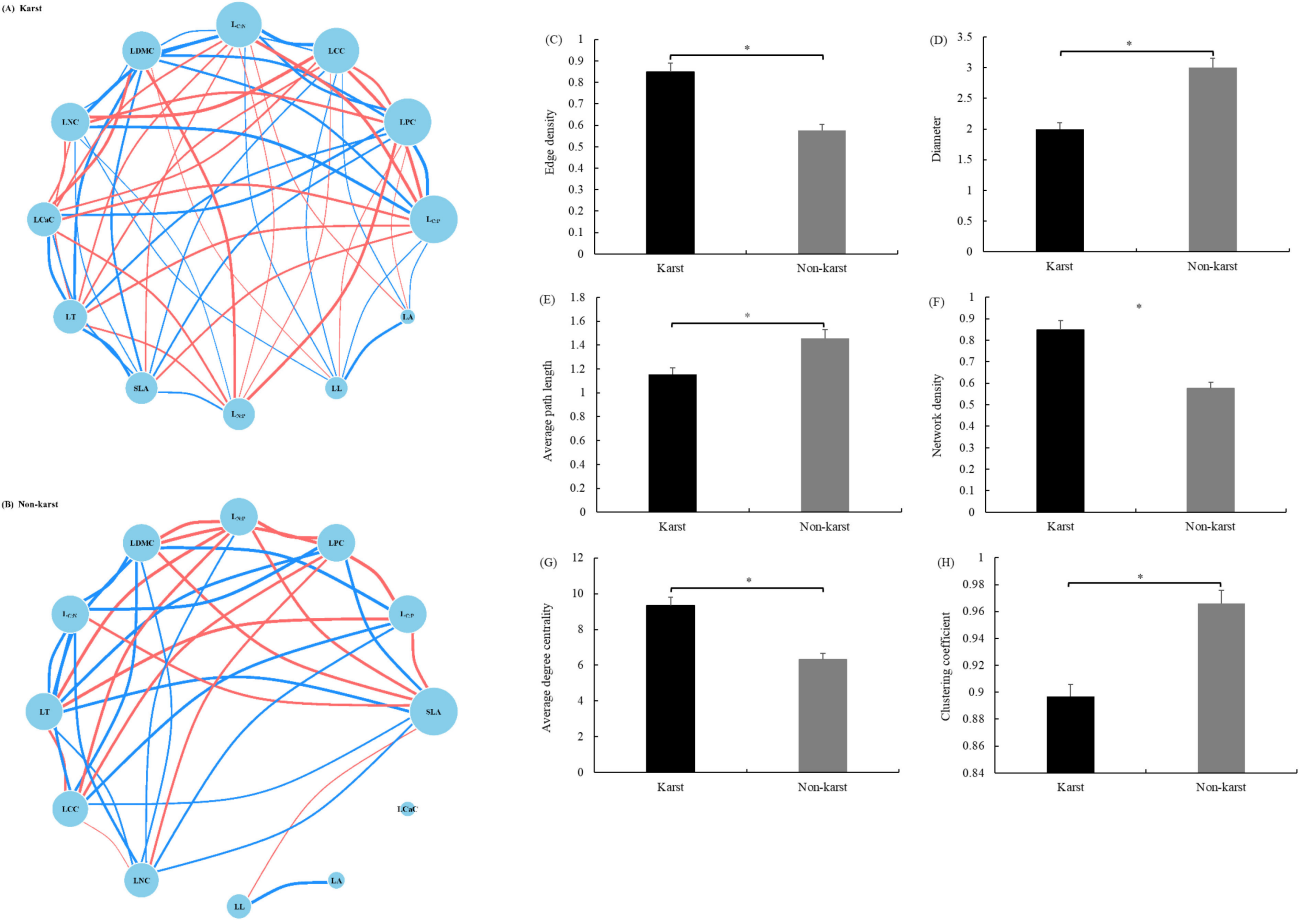
Leaf trait networks of *P. yunnanensis* in **(A)** karst and **(B)** non−karst forests. Nodes represent individual leaf traits. Red and blue lines represent positive and negative relationships, respectively. Line thickness is proportional to the absolute value of the correlation coefficient. Node size is scaled by degree centrality; larger nodes indicate hub traits with greater network connectivity. Panels **(C–H)** present quantitative comparisons of network topological metrics between the two habitats: **(C)** edge density, **(D)** network diameter, **(E)** average path length, **(F)** network density, **(G)** average degree centrality, and **(H)** clustering coefficient. Asterisks above bars indicate significant differences between habitats (Tukey's HSD test, *p< 0.05*).

Network analysis revealed a more interconnected leaf trait structure in karst habitats relative to non-karst habitats (**Figure 4**). The trait network under karst conditions exhibited a significantly smaller diameter, a shorter average path length, and a lower clustering coefficient, but a higher edge density, network density, and average degree centrality. In this network, the traits L_C:P_, LPC, LCC, and L_C:N_ displayed high values of degree, closeness, and betweenness centrality, identifying them as central traits. In the non-karst habitat network, however, SLA emerged as the central trait, demonstrating high degree, closeness, and betweenness centrality, whereas LCaC remained unconnected to other traits.

### 3.3 Linkage patterns between soil environment and leaf traits

**Figure 5:**
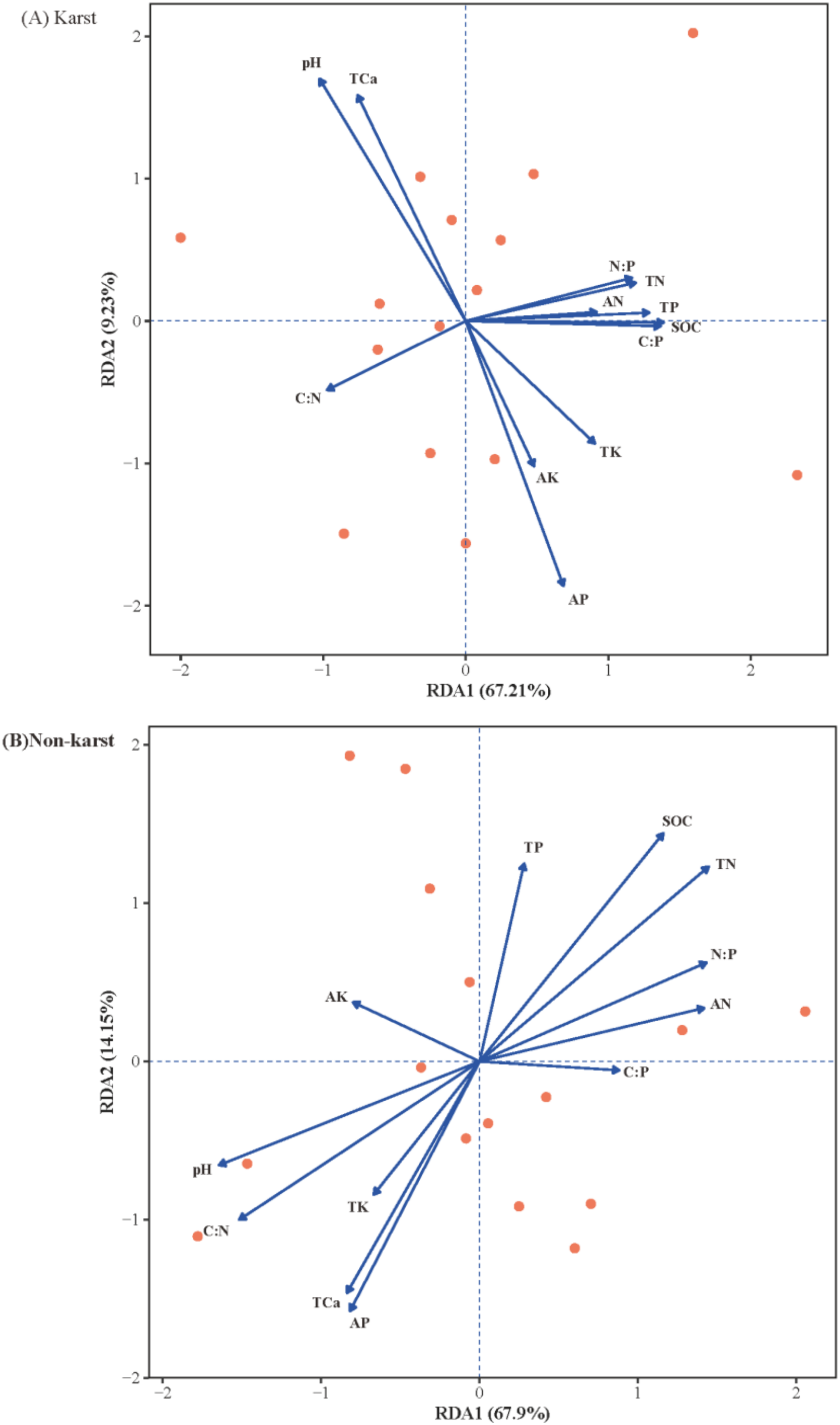
Redundancy analysis (RDA) ordination plots illustrating the relationships between leaf functional traits and soil physicochemical properties in **(A)** karst and **(B)** non−karst habitats.

Redundancy analysis (RDA) revealed significant differences in the environmental drivers of leaf functional traits between karst and non-karst habitats (**Figure 5**). In karst habitats, the first two RDA axes accounted for 76.44% of the trait variation (**Figure 5A**). RDA1 (67.21%) represented a strong soil phosphorus and stoichiometry gradient, showing positive correlations with soil TP, SOC, and C:P ratio. This axis clearly segregated trait combination of high LNC and LPC from those with high LCC and low LC:N, indicating that plant nutrient balance is predominantly controlled by soil phosphorus status. Notably, the significant drivers were confined to soil stoichiometric ratios (C:N, C:P, N:P) (*p< 0.01*), rather than individual nutrient concentrations or pH, highlighting the paramount importance of nutrient balance (stoichiometry) in karst habitats.

In contrast, the soil-trait relationships in non-karst habitats followed a different mechanism. The first two RDA axes collectively explained 82.05% of the total leaf trait variation (**Figure 5B**). The first axis (67.9%), representing a soil pH-nitrogen availability gradient, was positively associated with LDMC, LT, and LCC, but negatively associated with SLA and LPC. This pattern reflects a shift along the plant economics spectrum from conservative to acquisitive strategies. The second axis (14.15%) mainly reflected gradients of SOC and TP, and was closely related to LL and LA. Monte Carlo permutation tests further indicated that soil pH, AN, AP, and soil stoichiometric ratios (C:N, C:P, N:P) were significant drivers of leaf trait divergence (*p<0.05*).

Red points represent response variables (leaf functional traits), and blue arrows represent explanatory variables (soil physicochemical properties). Arrow length reflects the explanatory power of each soil variable, and arrow direction indicates its correlation with the ordination axes. The percentages of constrained variance explained by the first two RDA axes are displayed on the axis labels.

### 3.4 Pathways of habitat influence on leaf trait variation

**Figure 6:**
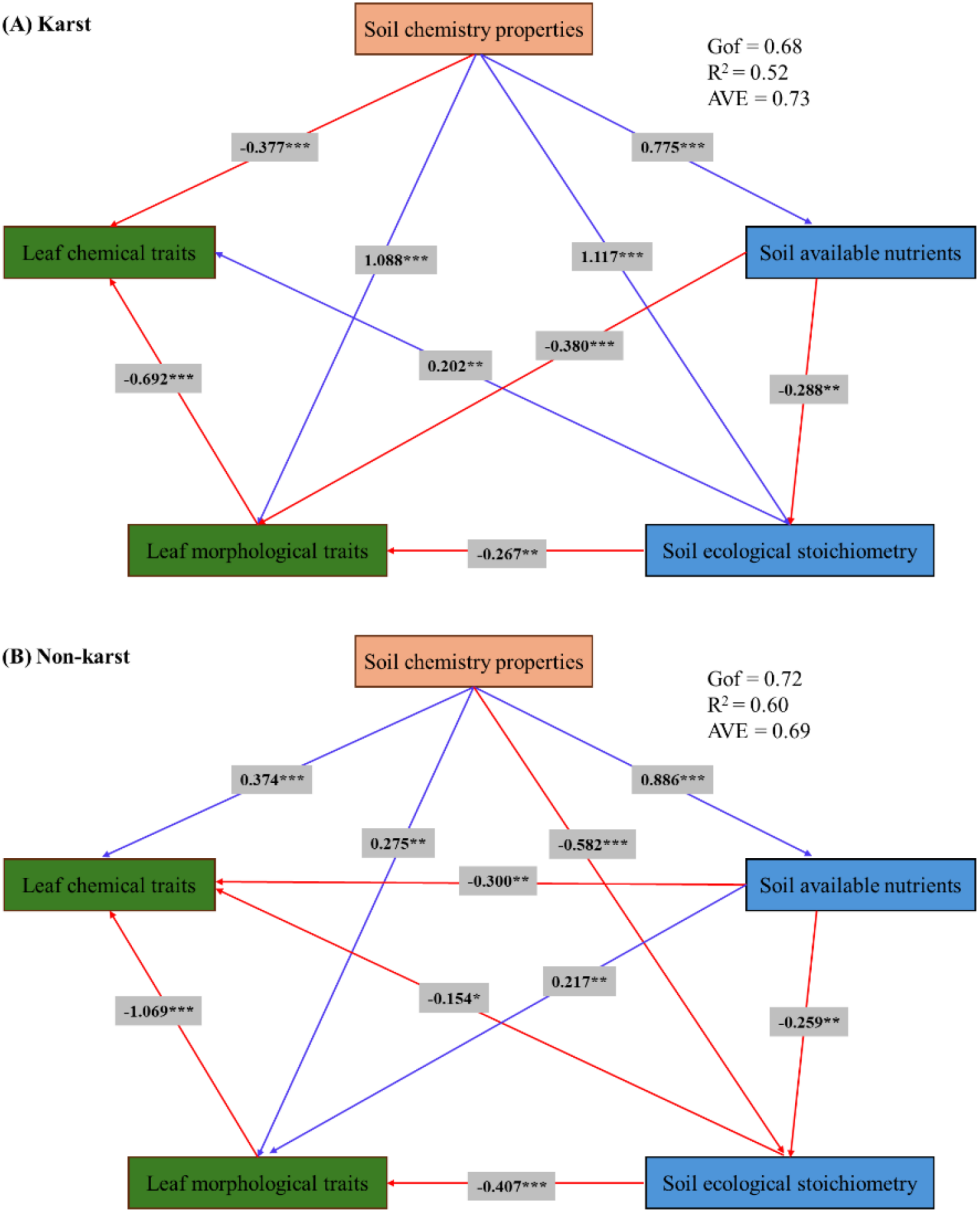
Partial least squares structural equation model results illustrating the effects of habitat on leaf functional traits in **(A)** karst and **(B)** non−karst habitats.

Path analysis via partial least squares structural equation modeling (PLS-SEM) quantified the direct and indirect effects of soil properties on leaf traits, revealing distinct habitat-dependent relationships (**Figure 6**). In karst habitats, the path relationships revealed greater complexity, characterized by a notable “promotion-inhibition” dichotomy (**Figure 6A**). Soil chemistry properties acted as the strongest positive driver of leaf morphological traits (path coefficient = 1.088, *p<0.001*) but exerted a direct inhibitory effect on leaf chemical traits (path coefficient = −0.377, *p<0.001*). A significant negative relationship was observed between leaf morphological traits and leaf chemical traits (path coefficient = −0.692, *p<0.001*), although weaker than observed in non-karst habitats. This suggests that under strong environmental filtering, trait trade-offs may be superseded by more critical survival strategies.

In non-karst habitats, soil chemistry properties exerted significant direct positive effects on both leaf morphological traits (path coefficient = 0.275, *p<0.01*) and leaf chemical traits (path coefficient = 0.374, *p<0.001*) (**Figure 6B**). Soil available nutrients also showed a positive direct influence on leaf morphological traits (path coefficient = 0.217, *p<0.01*). In contrast, a strong negative direct effect was observed from leaf morphological traits to leaf chemical traits (path coefficient = −1.069, *p<0.001*).

Blue arrows denote significant positive path coefficients, and red arrows denote significant negative path coefficients. Standardized path coefficients are displayed adjacent to each arrow. Color coding: Orange boxes represent soil chemical properties; light blue boxes represent soil ecological stoichiometry and available nutrients; dark green boxes represent leaf morphological traits and leaf chemical traits. Observed variables are shown in white boxes. Coefficients of determination (R²), Average Variance Extracted (AVE) and the global goodness−of−fit (GoF) index are displayed within the figure.

## 4 Discussion

### 4.1 Divergent plant adaptation strategies in karst and non-karst habitats

Functional traits often reflect the adaptation of plants to their environments (Li et al., 2025; Islam et al., 2024). The present study demonstrates significant differentiation in leaf functional traits of *P. yunnanensis* between karst and non-karst habitats (**Figure 2**). In karst habitats, a suite of coordinated changes in leaf functional traits was observed: LA, LL, and SLA were significantly lower, whereas LT and LDMC were markedly higher. This trait syndrome clearly indicates a shift in resource-use strategy from resource acquisition to resource conservation (Reich, 2014). Such morphological adjustments, characterized by the construction of tougher and longer-lived leaves, effectively reduce transpirational water loss (Liu et al., 2019) and enhance resistance to physical stress (Liu et al., 2020; Reich et al., 2003). Consequently, these adaptations improve plant tolerance to the combined stresses of drought and nutrient scarcity, facilitating survival in the arid and infertile conditions typical of karst ecosystems (Ordoñez et al., 2009).

The observed strategic shift is likely driven primarily by the prevalent phosphorus limitation in karst ecosystems (Lu et al., 2023). We found that karst soils had 33.72% lower available phosphorus content than non-karst soils (*t-test, p< 0.05*), while soil calcium content was 6.90 times higher in karst habitats (*p< 0.001*). These stark contrasts in soil chemistry were mirrored in leaf nutrient profiles. Specifically, LPC was 27.45% lower in karst populations, whereas LNC was unexpectedly 2.31% higher. Consequently, the L_N:P_ ratio increased from 10.979 in non-karst individuals to 15.454 in karst individuals, and the L_C:P_ ratio increased from 517.963 in non-karst individuals to 727.070 in karst individuals. This stoichiometric imbalance provides a mechanistic explanation for the morphological adjustments described above. Under phosphorus-limited conditions, the increase in LNC likely represents a compensatory mechanism to maintain photosynthetic capacity per unit leaf mass when leaf construction costs are high (Wright et al., 2004). This pattern—high leaf N coupled with low leaf P—has been documented as a specific adaptation syndrome in karst floras compared to adjacent non-karst vegetation (Liu et al., 2022; Zhang et al., 2018), and our findings confirm that *P. yunnanensis* adheres to this cross-species trend.

Notably, LCC and L_C:N_ remained stable across habitats, underscoring fundamental constraints in plant carbon economy. Despite the dramatic shifts in leaf N, P, and Ca concentrations documented above, our measurements showed no significant difference in leaf carbon concentration between karst and non-karst populations (p = 0.062). Similarly, the ratio of carbon to nitrogen remained unchanged (p = 0.9). This illustrates the hierarchical nature of trait adaptation, where plants maintain certain physiological relationships while modulating other traits to optimize fitness under local conditions.

In summary, the trait divergence observed in *P. yunnanensis* represents an integrated adaptation to the karst environment, not a set of independent responses. Under the strong environmental filtering imposed by phosphorus deficiency and drought stress, plants sacrifice rapid growth potential in favor of enhanced survival capacity (Kraft et al., 2015). This classic trade-off strategy underscores the pivotal role of soil phosphorus availability as a key driver in the functional ecology of karst ecosystems. Our data clearly indicates that the 33.72% reduction in soil available phosphorus is the primary environmental filter driving this strategic trade-off, a conclusion supported by the strong positive correlation between soil P availability and leaf SLA observed in our redundancy analyzes.

### 4.2 Effect of lithology on trade-offs in leaf functional traits

Functional correlations among plant functional traits exist, which represent the fundamental trade-offs among these traits (Westoby and Wright, 2006). Our findings further reveal that the variation in leaf functional traits of *P. yunnanensis* between karst and non-karst habitats extends beyond differences in trait means to encompass the coordination and integration among traits. This reorganization of trait relationships illustrates the role of environmental filtering in shaping plant phenotypic architecture (Pigliucci, 2003; Messier et al., 2017).

In our trait network analysis, the non-karst network was characterized by low edge density (0.576) and a central role for SLA (degree centrality = 9), whereas the karst network exhibited 47.22% higher edge density (0.848) and a shift in centrality toward stoichiometric ratios. In the more favorable non-karst habitats, the trait network was centered on SLA, consistent with the global leaf economics spectrum (Wright et al., 2004). SLA was positively correlated with LNC and negatively correlated with LT, LDMC, and L_N:P_, reflecting a typical resource-acquisition strategy (Reich, 2014). Within this configuration, LCaC occupied a peripheral position in the network, indicating its limited role in trait integration under low calcium stress (Wright et al., 2004). The observed weak trait relationships in non-karst forest species suggest weaker functional trade-offs. This condition fosters a wider range of viable trait combinations, which facilitates niche partitioning and ultimately supports species coexistence in relatively resource-rich environments (Markesteijn et al., 2011).

In contrast, under the high-stress conditions of karst habitats, the trait network was reorganized around phosphorus use efficiency and structural allocation, forming a more tightly integrated structure centered on LPC, L_C:P_, L_N:P_, and LCC. LCaC was not only significantly correlated with LPC, L_C:P_, L_N:P_, and LCC, but also positively associated with structural traits (LT and LDMC) and negatively with SLA, LNC, and LPC, suggesting a key role for calcium in the conservative phenotype, potentially mediating leaf construction and phosphorus starvation response (Wang et al., 2023). Given the enrichment of soil calcium we observed in karst sites, the elevated connectivity of leaf calcium within the trait network likely arises because plants must simultaneously regulate calcium uptake (to avoid toxicity) and phosphorus acquisition (to overcome deficiency). This dual constraint forces a tighter integration of calcium homeostasis with both structural reinforcement and nutrient metabolism, a linkage that is not present in the low-calcium, non-karst context. Quantitative analysis further indicated that the leaf functional trait network in karst habitats exhibited higher connectivity, edge density, and average degree centrality, along with shorter average path length, indicating a more constrained and integrated trait system (Messier et al., 2017). This tightly coupled structure may enhance phenotypic stability and facilitate coordinated responses to drought and nutrient stress, enabling efficient propagation of adjustments in individual traits through the network to maintain homeostasis. These results are consistent with previous findings that resource-limited environments exert strong selective pressures on plant species, leading to constrained trait evolution through tighter coordination and/or trade-offs (Flores-Moreno et al., 2019; Nardini et al., 2012).

In summary, these results demonstrate that habitat differences drive a strategic shift in *P. yunnanensis* from an SLA-centered, resource-acquisitive phenotype to a stoichiometry-centered, constrained phenotype emphasizing survival and resource conservation. Thus, adaptation to extreme environments involves not only adjustments in independent traits but also the evolution of integrated phenotypic modules, where the inter-dependencies among traits themselves may become targets of natural selection.

### 4.3 Driving mechanism of soil environments on leaf functional traits across different habitats

Our results demonstrate fundamental shifts in both key soil drivers and adaptive strategies of leaf functional traits in *P. yunnanensis* between karst and non-karst habitats, illustrating how environmental filtering operates through distinct biogeochemical pathways (Kraft et al., 2015).

Using redundancy analysis (RDA), we identified that soil pH explained 21.75% of the variance in leaf traits in non-karst habitats (*p<0.001*), whereas in karst habitats, soil stoichiometry (C:N:P ratios) emerged as the primary predictor, accounting for 24.87% of trait variation (*p<0.001*). In non-karst habitats, soil pH emerged as the most critical filter shaping leaf trait composition, a pattern consistent with observations in other ecosystems such as tropical and subtropical forests (Liu et al., 2022). By regulating microbial activity, nutrient availability, and heavy metal toxicity, soil pH constitutes a key physio-ecological filter that comprehensively influences plant nutrient acquisition and metabolic strategies (Kochian et al., 2015). In this study, lower soil pH was associated with higher SLA and LPC, suggesting an adaptive strategy that prioritizes resource capture and metabolic adjustment in acidic soils (Liu et al., 2022). In karst habitats, however, soil stoichiometry (C:N:P ratios) replaced pH as the primary driver of leaf trait variation—a direct consequence of the severe phosphorus limitation and high calcium loading inherent to carbonate-derived soils, as verified by our comparative soil analysis. Karst soils are generally characterized by high calcium and magnesium content, alkaline conditions, and extreme scarcity of available phosphorus (Chen et al., 2018). Under such conditions, elemental balance serves as a more meaningful ecological indicator than individual nutrient concentrations and exerts stronger selective pressure on plant phenotypes (Elser et al., 2007), thereby driving functional reorganization centered on stoichiometric optimization.

These differences in abiotic drivers correspond to fundamental shifts in plant strategy. In non-karst systems, trait variation aligns with the global leaf economics spectrum (Wright et al., 2004), reflecting continuous variation in resource investment strategies. In karst systems, however, leaf trait variation is more strongly associated with plant adaptation to phosphorus scarcity. The positive correlation between LPC and soil total phosphorus, along with the negative correlation between L_N:P_ and soil available phosphorus, suggests a “luxury consumption” strategy. Under extreme phosphorus limitation, plants may absorb and store scarce available phosphorus in leaves (“high accumulation, low utilization”) as a buffer against future phosphorus stress (Veneklaas et al., 2012). Furthermore, the close clustering of morphological traits (SLA, LA) and chemical traits (LNC, LPC) in ordination space indicates that leaf morphology and chemistry are co-selected and exhibit integrated adaptation under strong nutrient limitation.

Partial least squares path modeling (PLS-PM) analysis revealed a significant negative path from morphological to chemical traits in both habitats (non-karst: β = -1.069, *p<0.001*; karst: β = -0.692, *p<0.001*), corroborating the theory of inherent trade-offs in plant functional traits. The magnitude of this trade-off was, however, 35.27% weaker in karst habitats. This suggests that plants in more favorable environments retain greater phenotypic plasticity, which accentuates the trade-off between structural investment and chemical metabolism (Nicotra et al., 2010). This pronounced trade-off embodies a fundamental resource allocation conflict in leaves, representing a key intrinsic mechanism governing trait phenotypic expression in these habitats. In stark contrast, the severe phosphorus limitation characteristic of karst habitats appears to favor the development of an integrated phenotype, where key traits are co-selected and function synergistically, thereby attenuating the apparent strength of such trade-offs (Messier et al., 2017). This “canalization” effect mitigates internal trade-off intensity, indicating a strategic shift from a plasticity-dominated strategy toward a constraint-led and integration-oriented strategy — a crucial insight for predicting plant responses to environmental change. In karst habitats, soil chemical properties were the strongest positive driver of leaf morphological traits (β = 1.088, *p<0.001*), but exerted a direct negative effect on leaf chemical traits (β = -0.377, *p<0.001*). This pattern highlights a critical adaptive dilemma: while soil conditions promote morphological development, high concentrations of calcium and magnesium ions - likely through ion antagonism or physiological constraints - inhibit nutrient acquisition and accumulation in leaves. Within this constrained environment, soil ecological stoichiometry served as a significant positive pathway sustaining leaf chemical traits (β =0.202, *p<0.01*), underscoring the pivotal role of elemental balance in mitigating nutrient limitation.

While this study provides a comprehensive analysis of leaf functional trait differentiation and soil drivers in karst versus non-karst habitats, several limitations should be acknowledged. First, the limited number of plot replicates (n = 5 pairs) and the relatively low number of trees sampled per plot reduce the statistical power to detect intraspecific variation in leaf traits and subtle differences in secondary traits. Consequently, for those traits that did not exhibit significant differences between habitats, the current negative results may reflect Type II statistical error and should not be equated with a true absence of biological differences between the two habitats. Second, the sampling scope of this study was restricted to Weining County, Guizhou Province. Although this area is typically representative of the karst plateau surface spanning eastern Yunnan and western Guizhou, caution is required when directly extrapolating the conclusions to other climatic zones or to distinct karst landform types, such as peak-cluster depressions. Third, the investigation focused on a single species, *Pinus yunnanensis*, which, although widely distributed across the region, may not fully represent the diverse array of adaptation strategies exhibited by the entire karst flora. Nevertheless, the robust and significant habitat effects observed for key leaf morphological traits indicate that, after rigorous control for topographic factors, the distinctive water and nutrient stresses of the karst environment do indeed exert a dominant filtering effect on plant leaf construction. Future research that incorporates a larger number of plot pairs and a broader suite of species will be essential to validate the regional-scale generality of this geomorphological differentiation pattern and to deepen the mechanistic understanding of plant adaptation in karst ecosystems under global change.

## 5 Conclusion

This study demonstrates that karst habitats function as strong environmental filters that reshape both the mean values and the network architecture of leaf functional traits in *P. yunnanensis.* The main findings and their implications for ecological restoration are summarized as follows:

### 5.1 Trait shift toward resource conservation

In karst habitats, *P. yunnanensis* exhibited lower SLA and higher LDMC and LT, reflecting a strategic transition from resource acquisition to stress tolerance. Native species sharing this conservative trait syndrome are pre−adapted to phosphorus−poor, drought−prone karst soils and should be prioritized in restoration planting.

### 5.2 Reorganization of trait networks under stress

The karst trait network displayed 47% higher edge density and a shift in hub identity from SLA to LPC and stoichiometric ratios (L_C:P_, L_N:P_), indicating tighter phenotypic integration and canalization under environmental constraints. Network metrics such as edge density and hub identity may therefore serve as sensitive indicators of restoration progress, with tighter integration signaling recovery toward a stress−tolerant karst phenotype.

### 5.3 Primacy of phosphorus stoichiometry over pH

Soil C:N:P stoichiometry, rather than pH, was the dominant driver of leaf trait variation in karst habitats, underscoring the central role of phosphorus limitation in carbonate−derived soils. Restoration practices should consequently prioritize alleviating phosphorus deficiency — e.g., via mycorrhizal inoculation or slow−release phosphorus amendments — rather than focusing on pH modification.

Collectively, these findings advance beyond traditional trait−mean comparisons by demonstrating that karst adaptation involves a fundamental reorganization of trait network architecture and a shift in hub identity toward stoichiometric regulation. The findings establish a trait−based framework that links functional network metrics to practical restoration guidance, offering novel benchmarks for species selection and recovery monitoring in ecologically fragile karst landscapes. Future work should incorporate belowground traits and microbial processes to construct a more comprehensive plant–soil feedback network under global change.

## Data Availability

The raw data supporting the conclusions of this article will be made available by the authors, without undue reservation.
